# Bioavailability Enhancement of Paclitaxel via a Novel Oral Drug Delivery System: Paclitaxel-Loaded Glycyrrhizic Acid Micelles

**DOI:** 10.3390/molecules20034337

**Published:** 2015-03-06

**Authors:** Fu-Heng Yang, Qing Zhang, Qian-Ying Liang, Sheng-Qi Wang, Bo-Xin Zhao, Ya-Tian Wang, Yun Cai, Guo-Feng Li

**Affiliations:** Department of Pharmacy, Nanfang Hospital, Southern Medical University, Guangzhou 510515, China; E-Mails: fhyang2011@126.com (F.-H.Y.); zq1699@126.com (Q.Z.); qianyinglny@163.com (Q.-Y.L.); wsq2011@126.com (S.-Q.W.); zhaobx@smu.edu.cn (B.-X.Z.); alicemagic@126.com (Y.-T.W.); cayun2@126.com (Y.C.)

**Keywords:** glycyrrhizic acid, micelles, oral bioavailability, paclitaxel

## Abstract

Paclitaxel (PTX, taxol), a classical antitumor drug against a wide range of tumors, shows poor oral bioavailability. In order to improve the oral bioavailability of PTX, glycyrrhizic acid (GA) was used as the carrier in this study. This was the first report on the preparation, characterization and the pharmacokinetic study in rats of PTX-loaded GA micelles The PTX-loaded micelles, prepared with ultrasonic dispersion method, displayed small particle sizes and spherical shapes. Differential scanning calorimeter (DSC) thermograms indicated that PTX was entrapped in the GA micelles and existed as an amorphous state. The encapsulation efficiency was about 90%, and the drug loading rate could reach up to 7.90%. PTX-loaded GA micelles displayed a delayed drug release compared to Taxol in the *in vitro* release experiment. In pharmacokinetic study via oral administration, the area under the plasma concentration-time curve (AUC_0→24 h_) of PTX-loaded GA micelles was about six times higher than that of Taxol (*p* < 0.05). The significant oral absorption enhancement of PTX from PTX-loaded GA micelles could be largely due to the increased absorption in jejunum and colon intestine. All these results suggested that GA would be a promising carrier for the oral delivery of PTX.

## 1. Introduction

Paclitaxel (PTX) is a natural terpenoid compound, extracted from the bark of Western *Taxus brevifolia*, that has excellent antitumor activities against a wide range of solid tumors, including refractory human ovarian, breast cancers and non-small-cell lung carcinoma. This natural compound lacks ionizable functional groups, and thus exhibits poor aqueous solubility (<1 μg/mL) [1]. Clinically, PTX is administered by intravenous injection (I.V.) because of its low oral bioavailability (<2%) [2], which is resulted from the poor solubility, low permeability restricted by P-glycoprotein (P-gp) [3], and the metabolism by P_450_ enzymes like CYP3A4 [4].

The current formulation of PTX in common use, with the trade name Taxol^®^, is dissolved in a 50:50 (*v*/*v*) mixture of Cremophor^®^ EL/dehydrated ethanol. However, side effects caused by the addition of Cremophor^®^ EL including nephrotoxicity, neurotoxicity and cardiotoxicity have greatly limited its use [5]. In addition, Cremophor^®^ EL can also induce systemic and hematological toxicity through an oxidative stress-based mechanism, which is mainly responsible for the oxidative damage [6]. In addition, compared with injection, oral administration is a far more preferable option because of its better patient compliance, more economic and chronic treatment regimen [7]. Therefore, it is urgent to develop a new oral formulation to avoid the severe side effects without obvious reduction in the drug efficacy of PTX.

Several tactics have been proposed to solve the problems mentioned above, such as micro-emulsion drug delivery systems [8], polymer nanoparticles [9,10], solid lipid nanospheres [11], PTX-loaded PEO–PPO–PEO micelles [12], PTX-loaded Pluronic/LHR mixed polymeric micelles [13], PTX-loaded nanosponges [14] and nanoemulsions [15]. However, these formulations may have many drawbacks, such as the high cost, disintegration before reaching to the target [16], unsuitability for oral administration, serious side effects, potential toxicities, limited loading capacities [17], lack of biofunctionality and mutability to environmental changes [18]. Most importantly, some materials in the various new dosage forms of PTX are still waiting for verification of their safety to human beings and the projects would be time-consuming and cost-needed. 

**Figure 1:**
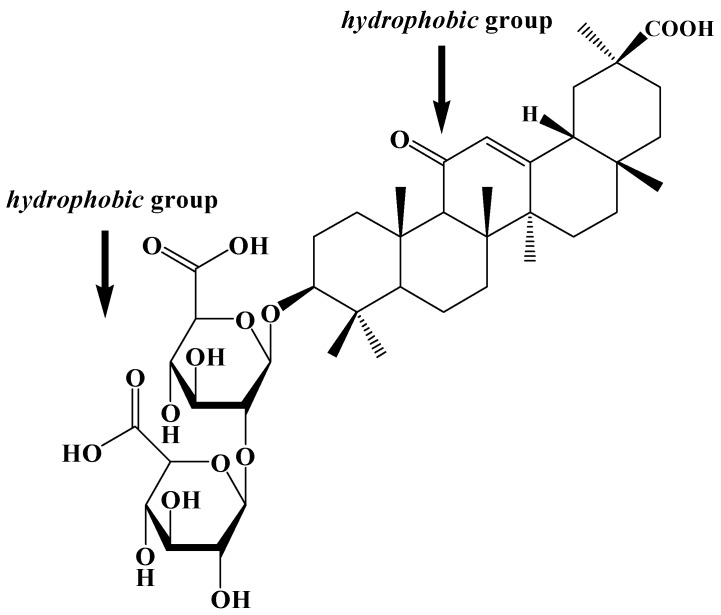
The chemical structure of glycyrrhizic acid.

Glycyrrhizic acid (GA), a triterpenoid saponin, is the major active compound of *Glycyrrhiza glabra* L, which is broadly used in medicinal herbs [19]. GA is found to have anti-inflammation activity, hepatoprotective effects and anti-tumor activity [20,21]. As a new carrier, GA is reported as a low-toxic oral absorption enhancer for hydrophobic drugs [21,22,23,24], which can increase not only the solubility of lyophobic drugs, but also their penetration through cell membranes as it is able to increase the permeability (about 60%) and decrease the elasticity modulus of cell membranes [21]. GA is also able to enhance the bioavailability of hydrophobic drugs in various ways. Chemically, GA is a conjugate of a glucose molecule and a glycyrrhetinic acid molecule. This triterpene glycoside demonstrates a capability of micelle-forming due to its amphiphilic structure (Figure 1). 

The concentration of GA and the acidity of solution may change its self-aggregation forms. At low concentrations (10^−5^–10^−3^ M), GA mainly forms dipolymer, whereas it forms large micelle-like aggregates at high concentrations (more than 10^−3^ M) with more than four molecules [25,26,27]. As a surfactant, GA aggregates or micelles can form the “host–guest” inclusion complexes with hydrophobic pharmaceutical compounds, which are capable of increasing the drug activity via enhancing the drug dissolution and preventing drug precipitation [27]. An *in vitro* study reported that GA inhibited the function of P-gp, in a similar way to glycyrrhetinic acid, the major metabolite of glycyrrhizic acid [28]. An *in-situ* research suggested that the absorption enhancement of aconitine could be achieved through the inhibition of P-gp by GA. When co-used with GA, the absorption enhancement of aconitine was remarkable in the distal intestine, where the expression of P-gp is higher than that of the other parts of intestine. In addition, GA could also increase the transport of digoxin, a substrate of P-gp, in rat ileum. However, its effects on the paracellular and transcellular pathways were slight. These phenomena indicate that GA is able to inhibit the activity of P-gp [23]. Moreover, it was also reported that GA was an inhibitor of CYP3A, CYP1A1, and CYP2E1 in rat liver microsomes [29]. Additionally, it was reported that the combination of GA brought notable therapeutic enhancement of several lyophobic compounds [23,30]. Some animal studies indicated that the complexes of tranquilizing drug phenibutum and GA showed an equivalent effect at a dose reduced by 16 times. Meanwhile, the toxicity of phenibutum decreased by a factor of 1.7 while the therapeutic index increased by a factor of 17 [30,31].

It is very interesting that both PTX and GA are regarded as terpene compounds. According to the rule of “like dissolves like” [32], GA may be able to dissolve PTX and increase its solubility. Especially, unlike many other new dosage forms of PTX, of which various materials were still unverified for clinical uses, because of their instability or unclear safety, GA has been used in clinic for years. The clear and predictable side effects of GA and its stability may be the advantages of this drug delivery system. Based on the above facts, it is assumed that GA maybe a promising carrier for enhancing both the solubility and the permeation of PTX and thus improving its oral bioavailability.

Hence, in the present work, we firstly investigated the interactions between PTX and GA and designed a nano-sized formulation that could largely increase the solubility of PTX using GA, a hypotoxic or even therapeutic material, as a carrier. Then, in order to characterize this formulation, the morphology, particle size, drug loading, zeta potential and release profile were investigated. Moreover, the oral bioavailability of this preparation and Taxol^®^ were compared to evaluate the effect of GA on enhancing the oral absorption of PTX *in vivo*.

## 2. Results and Discussions

### 2.1. Phase-Solubility Test

**Figure 2:**
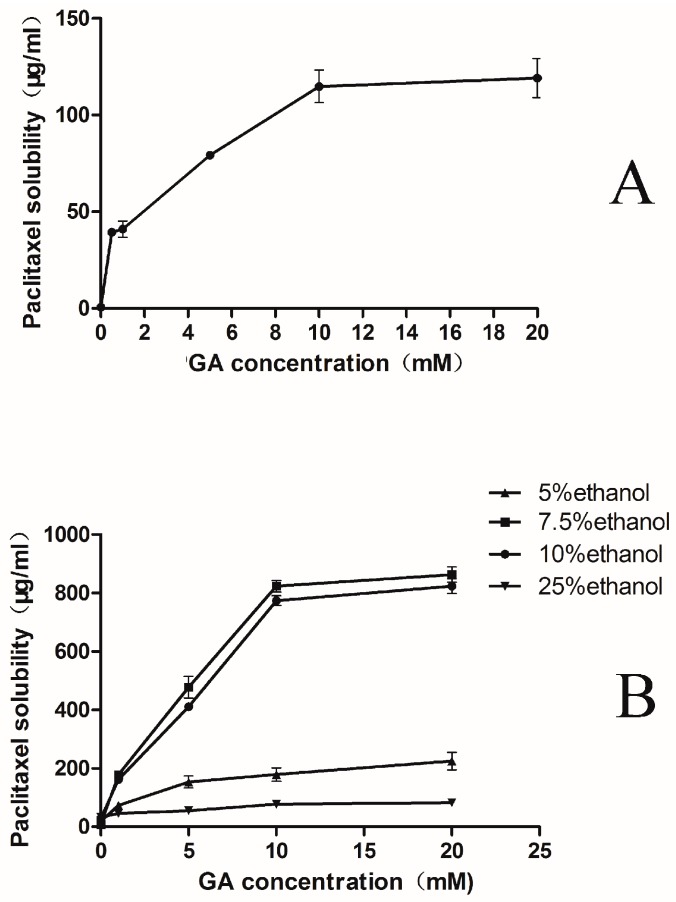

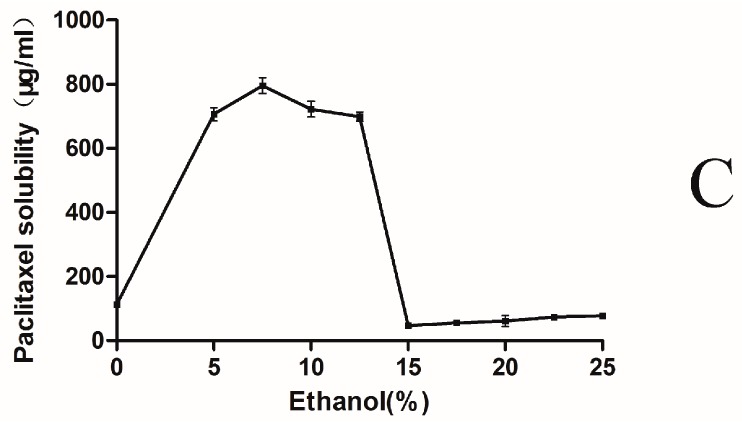
Phase-solubility diagram of PTX-GA systems at 25 °C in water (**A**), in different ethanol/water percentages solution(including 5%, 7.5%, 10% and 25% ethanol) (**B**), in different ethanol/water percentages with 10 mM GA (**C**), Data showed the amount of PTX solubilised as a function of the amount of GA added. The experiment was performed in triplicate (*n* = 3).

The phase-solubility chart for the complex formation between PTX and GA was shown in Figure 2A. This graph indicated that the solubility of the drug in water increased greatly as a function of GA concentration. The solubility of PTX in water, in the absence of GA, was merely about 0.67 ± 0.02 μg/mL, which was similar as the reported data [33]. However, the solubility increased approximate 200 folds (0.67→124 μg/mL) in the presence of 10 mM GA. When the concentration of GA increased up to 20 mM, the solubility of PTX changed little compared with 10 mM GA (Figure 2A). Moreover, the formulation containing 20 mM GA might easily transform into a GEL state. As a result, the highest concentration of GA used in this study was 10 mM. The exact value of critical micelle concentration for GA is 10^−3^ M [30]. At a certain concentration (10^−3^ M), GA mainly formed micelle-like aggregates [27], which were largely accounted for the solubility enhancement.

Based on the above results, a strategy of using ethanol as a co-solvent to further enhance solubility was adopted. The solubility tests of PTX with different percentages of ethanol in water (0%, 5%, 7.5%, 10% and 25%) were carried out. The results revealed that the solubility of PTX in different percentages of ethanol was no more than 30 μg/mL, but the solubility of PTX had a significant improvement on the basis of the addition of GA. With 10 mM GA, PTX presented the best solubility in the solution containing 10% ethanol (Figure 2B).

Interestingly, when the ethanol percentage reached up to 25%, the PTX solubility decreased sharply comparing with that in the 10% ethanol solution. It was reported that the addition of alcohol could significantly change the aggregation of micelles [34]. When the mole fraction of ethanol was higher than 0.04 in a binary mixture of ethanol and water, a continuous network of hydrogen bonds between ethanol and water were formed, which enhanced the solubility of PTX. When the mole fraction of alcohol equaled to 0.1, this structure reached maximum stability. As the concentration of alcohol further increased, the continuous network of hydrogen bonds started to disrupt. Therefore, the solubility of PTX decreased in 25% ethanol because of the resulted instability of micelles. In this regard, there should be an optimal percentage of ethanol that could effectually improve the solubility of PTX in the mixed solution.

To further investigate the effect of ethanol, phase-solubility test with an increasing ethanol percentage was carried out. Results showed the best percentage of ethanol was 7.5% (Figure 2C). However, the addition of 7.5% ethanol did not greatly enhance the solubility of PTX when GA was absent. According to the preliminary work, the PTX solubility could reach to 823.38 ± 14.41 μg/mL with the optimal ethanol percentage (7.5%), which was more than 1,000-fold higher (0.67→823 μg/mL) compared with the solubility of PTX in water. Thus, the micelle formulations could be prepared containing up to about 0.82 mg/mL PTX at a still reasonable GA concentration for clinical use (Figure 2B). The concentration of PTX was similar with that of the diluted Taxol^®^ in clinical use.

### 2.2. Characterization of the PTX-Loaded GA Micelles

The particle size and its distribution might be of great importance in determining the fate of micelles after oral administration. The micelle size and the size distribution were measured using dynamic light scattering (DLS) method, which was the most popular and common technique for determining the size distribution profile of nano-micelles in suspension [35]. The physiochemical parameters of the test samples (Table 1) showed that both bare and PTX-loaded micelles had narrow size distribution and small diameters. 

**Table 1:** Particle sizes, Zeta potential and polydispersity index values of the test micelles (*n* = 3).

Sample	Size (nm)	Zeta Potential (mV)	Polydispersity Index
Taxol^®^	15.1 ± 0.4	3.0 ± 0.2	0.333 ± 0.03
Bare GA micelle	82.3 ± 6.4	−10.0 ± 1.1	0.243 ± 0.03
PTX-loaded GA micelle	245.4 ± 5.6	−45.7 ± 1.8	0.192 ± 0.02

**Figure 3:**
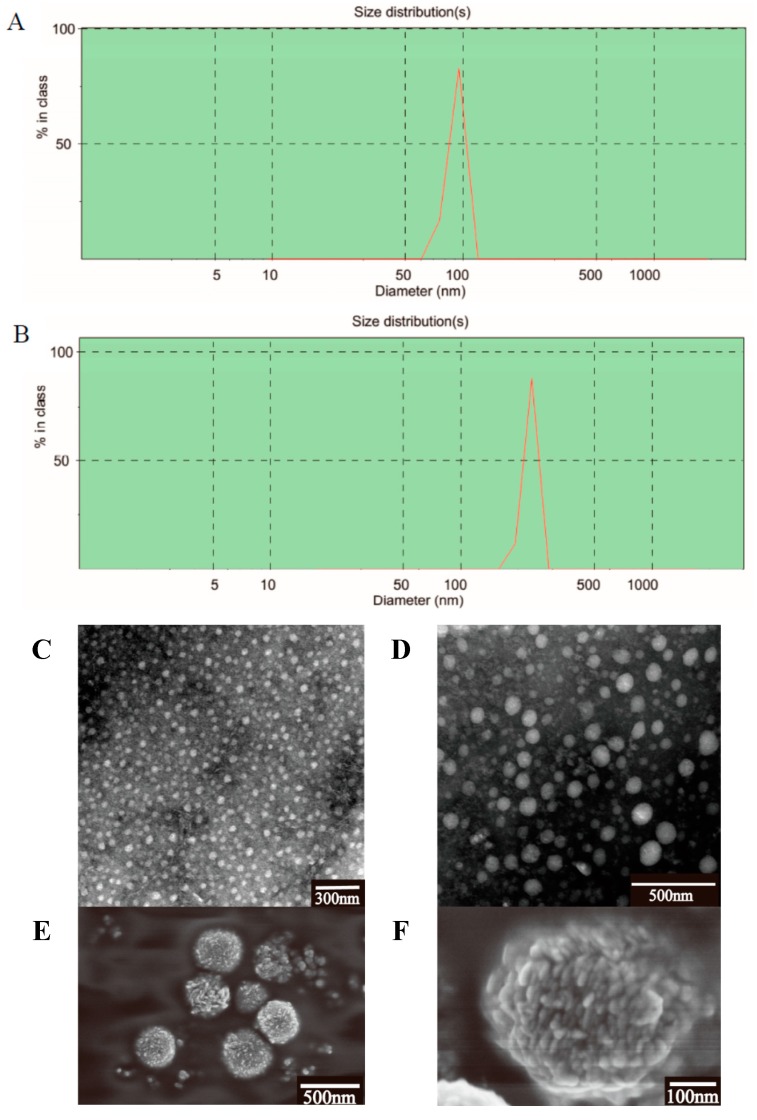
Malvern Zetasizer 3000 HSA size measurement of (**A**) blank micelles, (**B**) PTX-loaded GA micelles. TEM images of bare micelles (**C**) and PTX-loaded GA micelles (**D**). SEM images of PTX-loaded GA micelles (**E**, **F**).

Compared with the blank micelles (82.3 ± 6.4 nm), PTX-loaded micelles had a much larger average diameter (245.4 ± 5.6 nm), which suggested the drug was entrapped inside the micelles [36] (Figure 3A,B). Besides, its polydispersity indices (PDI) were found to be lower than 0.2, which was considered as an evidence of homogeneous micelles [37]. Additionally, the absolute value of the zeta potential of PTX-loaded micelles was 4.5-folds that of the bare ones. Zeta potential is a significant parameter representing the stability of micellar systems. It was reported that under a relatively high surface charge, particles could repel each other with a strong electrostatic repulsion force, thus improved the stability of the system [38]. In this test, the micelles displayed a zeta potential of about −45 mV, hence the PTX-loaded GA system was considered to be stable. The PTX-loaded GA micelles had a larger surface area than the blank GA micelles because of the entrapment of PTX molecules, which might be responsible for the enhancement of the negative charges on the micelles surfaces. 

The TEM results (Figure 3C,D) showed that all micelles were spherical in shape with good dispersibility. The TEM micrographs indicated that the micelles loaded with PTX had a larger size than the bare ones, which also suggested the incorporation of PTX into the GA micelles. It was observed that the micelle size measured by DLS method was a little larger than that obtained by TEM (Figure 3C,D). The inconsistency may be largely due to the difference between the dried state and the hydrated state [38]. However, this situation was still in accordance with the fact that the drug-loaded micelles became bigger than the blank ones.

The morphology of PTX-loaded GA micelles was also observed by scanning electron microscopy. It was reported that the SEM images of paclitaxel were in crystal state [39]. Several nearly sphere-shape and granular surface nano-micelles were formed, as observed by SEM, which could be attributed to the encapsulation of the GA materials (Figure 3E,F). The SEM images indicated the preparation existed as a form of nanomicelles. 

In addition, the encapsulation efficiency of PTX-loaded GA micelles was 90.22% ± 0.11%, while the drug loading rate was 7.90% ± 0.04%. The encapsulation efficiency was evaluated by ultrafiltration method, since the ultracentrifugation method could not separate the free PTX from the micelles. Generally, one molecule of the encapsulated compound was combined with four or more molecules of the micelle-forming compound to form micelles [25,26]. Thus the PTX-loaded GA micelles should have a molecular weight more than 3 kDa. Therefore, the ultrafiltration filters with 3 kDa molecular size-exclusion pores were chosen in this study. As a result, the free PTX was successfully separated and analyzed by HPLC via the ultrafiltration method. Methanol was chosen as the solvent to dissolve the drug loaded nano-micelles, because it could not only effectively destroy the nanomicelles and fully release the PTX, but also could dissolve the released PTX completely.

In the present study, we fixed the weight ratio of PTX and GA at 1:10 to obtain the formulation with the highest encapsulation efficiency, smallest particle size and lowest polydispersity index. This preparation also took the advantages of GA for both P-gp and CYP3A inhibitory and the high stability of GA micelles, while it displayed pharmaceutically acceptable physicochemical properties (polydispersity index < 0.2, the absolute value of Zeta potential > 45, particle size < 250 nm, encapsulation efficiency > 90%).

### 2.3. Micelles Stability

The storage stability of the PTX-loaded GA micelle solution was tested. The results showed that the micelles were stable when stored at 4 °C for at least 3 months. The particle size did not change obviously within the 3 months (*p* > 0.05), while the polydispersity index and the Zeta potential had a slight increase (*p* > 0.05), and the encapsulation efficiency declined (*p* > 0.05) (Table 2). These changes in 3 months were within 10%, suggesting that the PTX-loaded GA micelles were stable during the storage time period, which was largely due to the high absolute value of zeta potential. The stability study for a longer period was still under investigation.

**Table 2:** The storage stability of PTX-loaded GA micelles at 4 °C. Data were presented as the Mean ± S.D. (*n* = 3).

Time	Size (nm)	Polydispersity Index	Zeta Potential (mV)	Encapsulation Efficiency (%)
0 month	249.4 ± 4.5	0.181 ± 0.02	−45.7 ± 1.8	89.97 ± 0.82
1 month	247.7 ± 1.2	0.183 ± 0.02	−47.6 ± 2.7	87.64 ± 1.27
2 month	242.3 ± 9.0	0.187 ± 0.05	−48.9 ± 2.1	84.07 ± 3.24
3 month	227.8 ± 9.0	0.192 ± 0.03	−51.3 ± 3.2	82.57 ± 4.36

### 2.4. In Vitro Release

**Figure 4:**
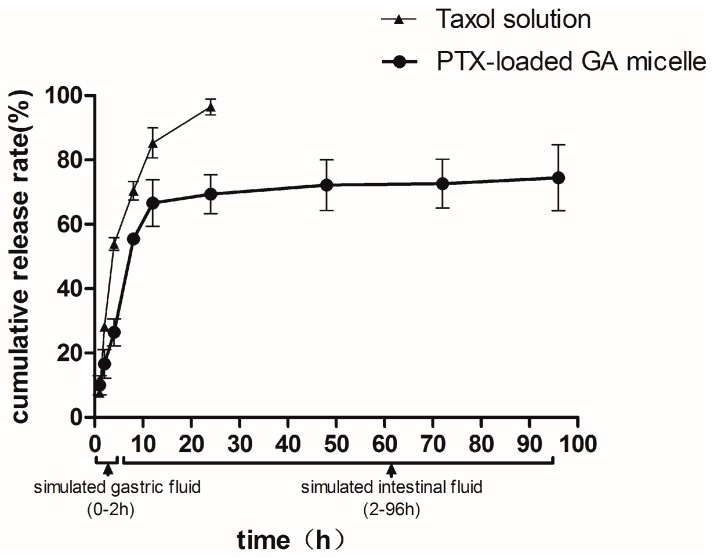
The cumulative release rates of paclitaxel from PTX-loaded GA micelles and Taxol^®^ (Cremophor EL-based formulation) as a function of time in simulated gastric fluid (0–2 h) and in simulated intestinal fluid (2–96 h) at 37 °C. Data were presented as mean ± S.D. (*n* = 3).

The *in vitro* release profile of PTX-loaded GA micelles was investigated in an aqueous medium containing Polysorbate 80 (1%, v/v) at 37 °C. The release rate of PTX-loaded GA micelles was less than 30% within the initial 2 h in SGF and the total amount of released PTX was about 70%–75% in SGF/SIF. The 12-hour cumulative release rates of GA micelles and Taxol^®^ were 66.62% ± 12.51% and 85.29% ± 4.65%, respectively. After 24 h, Taxol^®^ was almost completely released, showing an obviously faster release rate than the PTX-loaded GA micelles, which indicated that PTX-loaded GA micelle solution could be considered as a delayed drug release system compared to Taxol^®^ (Figure 4). 

GA should show its ability to resist fast release and degradation in harsh environment of GI tract, in order to ensure the delivery and absorption of the hydrophobic drugs loaded in the carrier. Besides, a rapid release would cause a drug precipitation in the aqueous fluids of the GI tract before absorption [40]. Thus, the delayed release property of PTX-loaded GA micelles could avoid rapid precipitation or leakage in the condition of GI lumen during the drug delivery and absorption of micelles. Furthermore, GA micelles might be able to protect the drug against gastrointestinal (GI) degradation.

### 2.5. DSC Analysis

**Figure 5:**
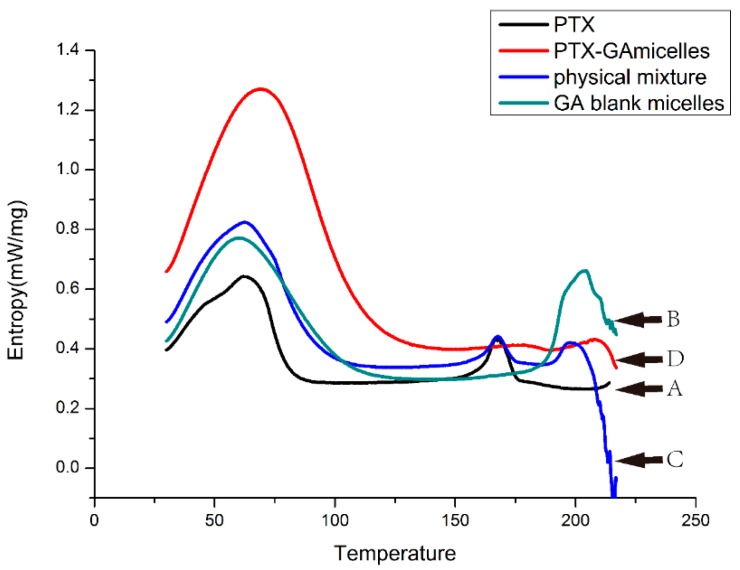
The DSC thermo gram of PTX powder (A), blank GA micelles freeze-dried powder (B), the physical mixture of PTX and blank GA micelles freeze-dried powder (C), and PTX-loaded GA micelles freeze-dried powder (D) from 0–220 °C.

To investigate the existing form of PTX in GA micelles, DSC analysis was conducted for PTX, blank micelles, the physical mixture of PTX and GA micelles, and PTX-loaded GA micelles. The DSC thermogram of PTX exhibited an endothermic peak at 168.3 °C, which was attributed to the melting of PTX. Blank micelles showed a sharp peak at 203.9 °C. For the physical mixture, all characteristic peaks of both components were presented with only a slight shift. The PTX-loaded GA micelles showed a similar curve to blank micelles, which indicated that PTX was entrapped and existed in an amorphous state in the GA micelles (Figure 5).

### 2.6. Pharmacokinetic Studies in Rats

**Figure 6:**
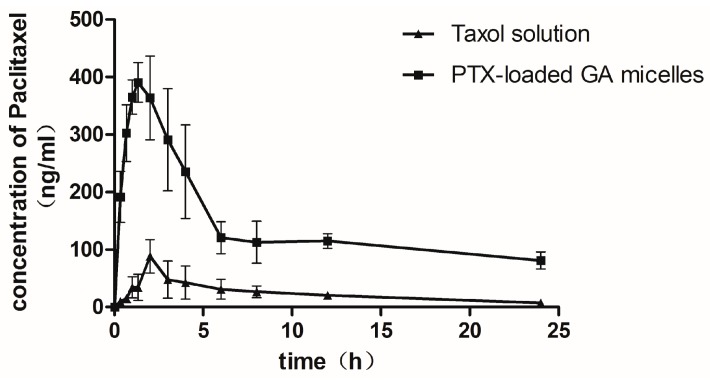
Mean plasma concentration-time profiles of paclitaxel following oral administration of paclitaxel at dose of 20 mg/kg of Taxol^®^ and PTX-loaded GA micelles. Each data presented as the Mean ± S.D. of 5 rats.

The oral absorption and dispositions of PTX from the Taxol^®^ and PTX-loaded GA micelle solution were studied in rats after administration of a single oral dose (20 mg/kg). The maximum concentration (C_max_) of PTX in PTX-loaded GA micelles (0.460 ± 0.10 μg/mL) was higher than that obtained in Taxol^®^ (0.095 ± 0.01 μg/mL). The area under the plasma concentration-time curve (AUC_0→24 h_) of PTX after oral administration of Taxol^®^ was 0.573 ± 0.12 μg·h/mL, whereas it was 3.42 ± 1.02 μg·h/mL for PTX-loaded GA micelle solution, which was six times greater than that of the Taxol^®^. The half-life of PTX in GA-loaded micelles (11.75 h) was higher than that in Taxol^®^ (9.49 h), although there was no significant difference between the two groups (Figure 6 and Table 3). 

It was reported that the restricted oral bioavailability of Taxol^®^ was not more than 2% [2]. The values of AUC_0→24 h_, C_max_, t_max_ and bioavailability of Taxol^®^ were similar to the reported pharmacokinetic parameters [13]. The data obtained from this study indicated that the PTX-loaded GA micelles led to a 6-fold enhancement in the oral bioavailability of PTX. The clearance of PTX-loaded GA micelles (1.09 ± 0.23 L/h) was six times less than Taxol^®^ (7.47 ± 0.98 L/h), while the half-life of PTX in GA micelles was slightly higher than that in Taxol^®^ (Table 3). It indicated that GA micelles were able to protect PTX from being cleared, which resulted in a relatively higher systemic exposure [8].

**Table 3:** Pharmacokinetic parameters of PTX after oral administration or I.V administration of Taxol^®^ (Cremophor^®^ EL-based formulation) and PTX-loaded GA micelles in rats (*n* = 5).

Parameters	Taxol (Oral)	PTX-Loaded-GA Micelle (Oral)	Taxol (I.V.)
C_max_ (μg/mL)	0.10 ± 0.01	0.46 ± 0.10 *	20.034 ± 5.701
t_max_ (h)	2.60 ± 0.89	1.63 ± 0.92	——
AUC_0–24 h_ (μg·h/mL)	0.57 ± 0.12	3.42 ± 1.02 *	10.26 ± 1.42
t_1/2_ (h)	9.49 ± 3.52	11.76 ± 5.76	12.33 ± 7.91
MRT(h)	8.18 ± 1.12	9.08 ± 1.34	3.56 ± 1.58
CL(L/h)	7.47 ± 0.98	1.09 ± 0.23 *	0.46 ± 0.10
Absolute bioavailability(%)	1.68	10.0 *	100
Relative bioavailability(%)	100	596.38	——

The dosage of PTX in oral administration was 20 mg/kg, and the I.V. dosage was 6 mg/kg. Date were expressed as the means of 5 rats S.D. * *p* < 0.05, compared with Taxol (Oral).

The above consequences might be largely due to the influence of GA, which effectively inhibited the drug efflux caused by the P-gp in intestine [23]. The interaction between P-gp and PTX was also avoided due to the entrapment in GA micelles. It was reported that the transport mechanism of micelles across intestinal mucosa might be achieved via the endocytic pathway, thus bypass P-gp efflux [13]. In addition, the inhibition of P_450_ enzymes, especially CYP_3_A_4_, by GA might also improve the intestinal absorption of PTX [29]. At first, we preferred to choose “Abraxane” as a control group. However, albumin, the protein drug carrier of “Abraxane”, exhibited gastrointestinal tract instability, which largely impeded the oral administration of “Abraxane”. Therefore, we did not choose “Abraxane” as a control group. What’s more, Taxol^®^ showed a much smaller particle size compared with PTX-loaded GA micelle (Table 1). Generally, the small particles (<100 nm) may result in low drug encapsulation and fast drug release. It had been proved that nanoparticles of approximate 200 nm in diameter have potential to deliver the drug across the GI barrier. The suitable diameters (250 nm) of PTX-loaded GA micelles might enhance the absorption of PTX [41,42].

In summary, this significant improvement of the pharmacokinetic parameters of PTX after oral administration of the PTX-loaded GA micelles could be explained by the comprehensive actions of the following effects: (1) the solubility enhancement of PTX by GA micelles; (2) As a nanosize drug delivery system (250 nm), it could reduce the uptake by mononuclear phagocyte system (MPS), allowing along circulation in the body [13], whereas whether the absorption was in the form of whole micelle needed further investigation; (3) The enhancement of PTX oral absorption could be partly due to the inhibitory effect of GA on intestinal P-gp [23,28]; (4) The reduction of the metabolic elimination of PTX in liver was possibly owing to the inhibition of CYP450 by GA [29]. 

### 2.7. Intestinal Absorption of PTX by in Situ Closed Loop Method

**Figure 7:**
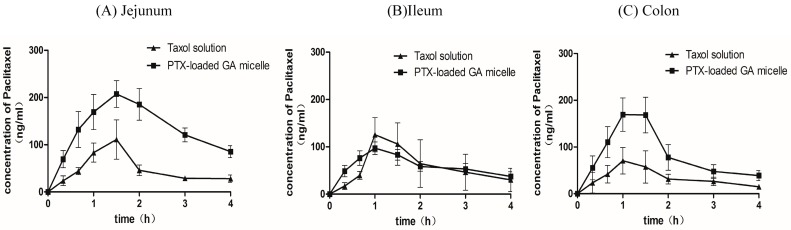
Mean plasma concentration-time profiles of paclitaxel following its administration into the jejunal loops (**A**), the ileal loops (**B**) and the colonic loops (**C**) of rats at a dose of 5 mg/kg of Taxol^®^ and PTX-GA micelles. Each data presented the mean ± S.D. of 5 rats.

The intestinal absorption of PTX from the Taxol^®^ and PTX-loaded GA micelle solution were studied in rats by an *in situ* Closed Loop Method. The inter-group comparison among the three regions of intestine showed that the area under the plasma concentration-time curve (AUC) obtained after the intestinal administration of the PTX-loaded GA micelles was about 2.7 folds increased compared to the Taxol^®^ in the jejunum region (*p* < 0.05). When it came to the colon region, the values of C_max_ and AUC in the PTX-loaded GA micelles group were about 2 fold more than those measured in the Taxol^®^ group (*p* < 0.05). These two formulations displayed no significant difference of AUC in the ileum region. In addition, the comparison among the three regions of intestine after intestinal administration of PTX-loaded GA micelles indicated that the AUC of the jejunum group was significantly higher than the ileum (*p* = 0.006) and colon groups (*p* = 0.026). PTX-loaded GA micelles showed the best absorption in jejunum among the three intestinal regions. Besides, there were no significant differences of AUC, C_max_ and t_max_ among the three regions of intestine after intestinal administration of Taxol^®^ (Figure 7 and Table 4).

This suggested that the significant increase of oral absorption of PTX from the PTX-loaded GA micelles could be largely due to the enhancement of the PTX absorption in jejunum and colon. The improved *in situ* closed loop models provided a novel application to determine the differences of the intestinal drug absorption in varied regions of intestine and from different formulations. When compared the intestinal absorption of PTX-loaded GA micelles with the Taxol^®^ in the same region of intestine, we found the AUC of PTX in the PTX-loaded GA micelles group was significantly higher than that of the Taxol^®^ group in the jejunum and colon regions (*p* < 0.05). In addition, we also found a regionally different effect of PTX-loaded GA micelles on the intestinal absorption of PTX. The AUC of PTX from the jejunum was relatively higher than the other intestine regions in the PTX-loaded GA micelles group (*p* < 0.05).

**Table 4:** Pharmacokinetic parameters of PTX after intestinal administration of Taxol^®^ (Cremophor^®^ EL-based formulation) and PTX-loaded GA micelles in different intestine regions determined by an *in situ* closed loop method (*n* = 5).

Parameters	C_max_ (μg/mL)	t_max_ (h)	AUC_0–4 h_(μg·h/mL)	t_1/2_ (h)
**Jejunum**				
Taxol^®^	0.12 ± 0.08	1.30 ± 0.27	0.19 ± 0.10	1.65 ± 0.48
PTX-loaded GA micelle	0.21 ± 0.06	1.60 ± 0.22	0.54 ± 0.20*	2.32 ± 1.33
**Ileum**				
Taxol^®^	0.23 ± 0.12	1.20 ± 0.27	0.23 ± 0.12	1.34 ± 0.57
PTX-loaded GA micelle	0.24 ± 0.10	1.10 ± 0.22	0.24 ± 0.10	2.48 ± 1.28
**Colon**				
Taxol^®^	0.08 ± 0.03	1.07 ± 0.48	0.14 ± 0.04	1.92 ± 0.79
PTX-loaded GA micelle	0.18 ± 0.03 *	1.20 ± 0.27	0.31 ± 0.13 *	1.73 ± 0.73

The dosage of PTX in intestinal administration was 5 mg/kg. Date were expressed as the means of 5 rats S.D. * *p* < 0.05, compared with Taxol^®^.

## 3. Experimental Section

### 3.1. Chemicals

Glycyrrhizic acid (GA) was purchased from Dalian Meilun Biotech Co., Ltd. (Dalian, China); Paclitaxel (PTX, purity > 99%) was obtained from the National Institutes for Food and Drug Control, Beijing, China. Docetaxel was purchased from Sigma-Aldrich Chemical Co. (Santa Clara, CA, USA). Sodium taurocholate and lecithin were purchased from Dalian Meilun Biotech Co., Ltd.. NaCl and KCl were also purchased from Dalian Meilun Biotech Co., Ltd. All other chemicals and solvents were analytical grade, except acetonitrile, which was HPLC grade.

### 3.2. Animals

Male Sprague-Dawley (SD) rats weighing between 200 and 250 g were supplied by the Laboratory Animal Center of Southern Medicine University (license: SCXK, Guangdong, 2006-0015) (Guangzhou, China). The rats were housed in a room under controlled temperature (20 °C to 24 °C), relative humidity (40% to 70%), and 12 h light/dark cycle. The animal experimental protocol was approved by the ethics committee of Southern Medicine University (No: 2011-0015, Date: 12 September 2014). All animal studies were carried out according to the guide for care and use of laboratory animals. The rats were fasted but allowed free access to water for 12 h before the experiment.

### 3.3. Methods

#### 3.3.1. Solubility Test

The phase-solubility test was performed with a previously reported method [43]. An excess quantity of PTX was taken into clean and dry vials containing 1 mL of various concentrations of GA solutions (0.01–10 M). Herein, clear and stable GA solution could be more easily prepared by solving in 70 °C deionized water. Since PTX had a better solubility in ethanol than in water, a certain amount of ethanol was also added as a hydrotropy agent in order to further increase the solubility of PTX. After sealed, these vials were shaken in a VorTemp 56 tm shaking incubator (Labnet International Inc., Edison, NJ, USA) at 25 °C for 48 h to achieve solubility equilibrium. Ultimately, the samples were filtered through 0.45 μm filters and the concentrations of PTX were detected by high performance liquid chromatography (HPLC; LC-20A, Shimadzu, Japan) (see Section 3.3.6.). All tests were repeated at least three times.

#### 3.3.2. Preparation of PTX-Loaded GA Micelle

The PTX-loaded GA micelles was prepared by the ultrasonic dispersion method, at the molar ratio of 1:10 (PTX:GA). Via single factor investigation, three critical influential factors (the ratio of drug to carrier, the pH of the solution and the percentage of ethanol) were found. The optimized preparation method was determined using an orthogonal design, and shown as follows: firstly, PTX (100 mg) was dissolved in ethanol (10 mL). Then, an aliquot of the solution (0.8 mL) was added into water (9.2 mL) containing GA (80 mg, containing 8% ethanol). The obtained mixtures were further dispersed using ultrasonic homogenizer (FS-600, Shengxi Experiment Instrument Co., Ltd., Shanghai, China, 600 W output) with the amplitude set at 40% of the maximum for 30 min in an ice bath, and the stable PTX-loaded GA micelle solution was made.

#### 3.3.3. Particle Size, Size Distribution and Zeta Potential

Particle size, polydispersity index (PDI) and zeta potential were determined by a Malvern Zetasizer 3000 HSA (Malvern, Worcs, UK). The prepared PTX-loaded GA micelles were diluted to a suitable concentration (10-fold dilution) with distilled water, and the samples were tested immediately. Experiments were conducted in triplicate.

#### 3.3.4. Transmission Electron Microscopy (TEM) 

The PTX-loaded GA micelles were negatively stained with phosphotungstic acid and observed under TEM. Briefly, a drop of micelle solution was placed on a copper grid, the excess liquid was drained with a filter paper, and the grid was dried at room temperature. The copper grid was stained in a 2% phosphotungstic acid solution (pH = 7.0) for 2 min. Observations were performed at 80 kV with a transmission electron microscope (Hitachi 7650, Tokyo, Japan).

#### 3.3.5. Scanning Electron Microscopy (SEM) 

SEM images of PTX-loaded GA micelles were obtained using a Hitachi S-4800 scanning electron microscope (Tokyo, Japan) at an acceleration voltage of 15 keV and probe current of 25 mA. The samples with water content were freeze-dried (see Section 3.3.6.), and then plunged into liquid nitrogen for 5 min and dispersed into smaller pieces for analysis. Before SEM examination, they were sputtering coated (SC7640 Sputter, Yufeng Experiment Instrument Co., Ltd., Shanghai, China) with gold.

#### 3.3.6. Determination of the Drug Encapsulation Efficiency and Drug Loading Rate

The drug encapsulation efficiency was determined by an indirect method—Ultrafiltration method. The ultrafiltration method was used to separate the free PTX in micelle solution, which was less potential for micelle deformation and therefore less compromised to the integrity of micelle. The samples were centrifuged in ultrafiltration filters composed of regenerated cellulose with 3 kDa molecular size-exclusion pores (Millipore^®^, Darmstadt, Germany), and the filtrate was analyzed using a high-performance liquid chromatography (HPLC; LC-20A, Shimadzu, Kyoto, Japan) equipped with an ultraviolet detector (SPD-20A, Shimadzu Co.). PTX was chromatographed by injection of 20 μL sample into a C18 column (5 μm, 100 mm × 4.6 mm). The mobile phase was acetonitrile-water (50/50, *v*/*v*) running at a flow rate of 1.0 mL/min with an oven temperature of 30 °C and detected at 227 nm. The retention time of PTX was 10 min while the run time was 12 min for each sample. 

Total PTX (100%) in the micelle suspension was detected after diluting the suspension in methanol to dissolve the GA and completely release the PTX, which was quantified using a calibration curve.

The encapsulation efficiency (EE, %) was expressed as the ratio of the PTX amount encapsulated by the GA micelles and the total PTX (100%) amount in the micelle suspension, as described by Equation (1):

(1) $$\text{EE}\%=(1-\frac{\text{Wf}}{\text{Wtotal}})\times 100\%$$

where W_f_ was the weight of PTX in the filtrate, and W_total_ was the weight of PTX in the formulation.

10 mL of PTX-loaded GA micelle solution were dialyzed in deionized water for 2 h to remove the organic solvent and lyophilized for 24 h (FD-1B-50, Boyikang Experiment Instrument Co., Ltd., Beijing, China). Accurately weighed freeze-dried micelles (2 mg) were mixed with methanol (50 mL) dissolving the micelles by sonication (SB25-12YDTD, Scientz, Ningbo, China) for 2 h to fully release the PTX, the amount of which was determined by HPLC. The drug loading (DL, %) was then calculated as the ratio of the PTX amount encapsulated by the micelles and the total weight of the micelles and PTX, as described by Equation (2):

(2) $$\text{DL}\%=\frac{\text{Ws}}{\text{Wp}+\text{s}}\times 100\%$$

owhere Ws was the weight of PTX quantified in the micelles, and W_P+S_ was the total weight of the micelles and PTX.

#### 3.3.7. The Storage Stability of PTX-Loaded GA Micelle

To test the storage stability, the PTX-loaded GA micelle solution was placed in a 10 mL Eppendorf tube and stored at 4 °C for at least 3 months. Samples were taken to determine the particle size, the polydispersity index, the zeta potential and the encapsulation efficiency once a month. 100 μL of the sample was diluted into 1 mL with methanol and dissolved by ultrasonic for 1 h, then the diluted sample was filtered through a 0.45 μm filter and analyzed by HPLC (see Section 3.3.6.).

#### 3.3.8. *In Vitro* Release of Drug-Loaded Micelles Solution

The *in vitro* release study was performed to investigate the differences of release behaviors between the PTX-loaded GA micelles and the commercial Taxol formulation. The PTX-loaded GA micelle solution (2 mL) containing 1.6 mg PTX was added to a dialysis bag with a molecular weight cutoff of 5000 Da, tied and immersed into the release medium (100 mL) at 37 °C with uniform stirring. Simulated gastric fluid without pepsin (SGF, pH = 1.6; 0.01–0.05 mol/L HCl, 2 g NaCl, 1 L water) and simulated intestinal fluid without trypsin (SIF, pH = 6.5; 3 mM sodium taurocholate, 0.75 mM lecithin, 3.0 g K_2_HPO_4_, 7.7 g KCl, 1 L water) were used as release media, respectively. After 1 h incubation in 100 mL SGF, the sample bags were transferred into 100 mL SIF and incubated up to 96 h. Polysorbate 80 (1%, v/v) was also included in the release media as a PTX solubilizing agent to ensure the sink conditions [39]. At a specified time interval, an aliquot (1 mL) was withdrawn and replaced with an equal volume of the fresh solution (SGF or SIF). The PTX concentrations in the aliquots were determined by HPLC (calibration curve of free PTX was obtained from SIF, ranging from 3.906 to 500 ug/mL, the LOQ = 3.906 ng/mL, r^2^ > 0.999).

Taxol^®^ was prepared by dissolving 10 mg of PTX in ethanol with an equal volume of Cremophor^®^ EL, and then sonicated for 30 min [13,44], followed by 7-fold dilution to make an equivalent concentration of PTX-loaded GA micelles. The *in vitro* release of PTX from Taxol was determined following the same procedures as PTX-loaded GA micelles. All assays were performed in triplicate. The release profiles were expressed in terms of cumulative release in percentage, and plotted *versus* time:

$$Cumulative\;PTX\;released\%=\frac{\text{Ci}∙\text{V}+\text{Ve}(\text{Ci}-1+\text{Ci}-2+\text{Ci}∙\text{Ve})}{\text{W}}\times 100\%$$

where C_i_ represents the i th sampling concentration of PTX (μg/mL), V is the total volume of release medium (mL), V_e_ is the sampling volume (mL) and W is the total weight of PTX in the micelles (mg).

#### 3.3.9. Differential Scanning Calorimetry (DSC) Analysis

Four kinds of samples including PTX powder, freeze-dried powder of blank GA micelles, the physical mixture of PTX and the freeze-dried powder of blank GA micelles, and the freeze-dried powder of PTX-loaded GA micelles (without cryoprotectant) were weighted and sealed in the aluminum pans, and then scanned from 25 to 250 °C at a heating rate of 10 °C/min on the DSC 204 F1 System (Netzsch, Munich, Germany). Analysis was performed under a nitrogen gas atmosphere.

#### 3.3.10. Pharmacokinetic Studies in Rats

The pharmacokinetic test was carried out based on a reported method with modifications [45]. All animals used for this study were treated according to the protocols evaluated and approved by the South Medical University Animal Care and Use Committee. Ten male S.D. rats were housed individually under normal conditions and fasted overnight before experiment with free access to water They were randomly divided into two groups with five rats each and administered orally with two types of formulations: Taxol^®^ solution prepared in a 50/50 (*v*/*v*) mixture of Cremophor^®^ EL/dehydrated ethanol (containing 7.5% ethanol) and PTX-loaded GA micelles solution (containing 7.5% ethanol) at a PTX dose of 20 mg/kg, respectively. Blood (0.2 mL) was withdrawn from the subclavian vein at each given time intervals, then placed into heparinized tubes and separated immediately by centrifugation (4,000 rpm for 10 min). The obtained plasma was stored at −20 °C for future analysis.

Rat plasma (100 μL) was extracted with 2 mL mixed solvent (ethyl acetate: dichloromethane: acetonitrile = 4:1:1) after addition of 100 μL docetaxel (internal standard; 100 ng/mL). The mixture was vortexed for 2 min and then centrifuged at 15,000 rpm for 20 min. The supernatant was transferred to clean glass test tubes and dried in a vacuum drying apparatus (SalvisLab VC20 Vacucenter, Rotkreuz, Switzerland)). The residues were reconstituted with 100 μL of mobile phase and centrifuged at 15,000 rpm for 30 min, then analyzed by Agilent6460 Triple Quadrupole LC/MS system. Detection was performed with multiple reactions monitoring using electrospray ionization. The precursor/production transitions of paclitaxel and docetaxel (internal standard) at a collision energy (CE) of 22 eV were monitored at m/z 876.29→308.1 and 830.34→549.3, respectively. The fragmentor of paclitaxel and docetaxel (internal standard) were set at 250 eV and 210 eV. Separation of the analytes from plasma was achieved by using a Mediterranea Sea C18 analytical column (3.5 μm, 150 mm × 2.1 mm; Agilent Technologies, Inc., Santa Clara, CA, USA) with a flow rate of 0.3 mL/min at 40 °C. The mobile phase consisted of formic acid buffer (0.1% formic acid) and methanol (5:95, *v*/*v*). The retention time of paclitaxel and docetaxel (internal standard) were 1.243 min and 1.249 min, respectively. The ionization was conducted using an electrospray ionization interface in the positive mode. The drying gas temperature was maintained at 350 °C, at a flow-rate of 10 L/min with an ion spray voltage of 3.5 kV. The calibration curve of PTX in rat plasma was ranging from 5.5 to 550 ng/mL, the limit of quantification (LOQ) = 0.55 ng/mL, r^2^ > 0.999.

#### 3.3.11. Intestinal Absorption of PTX 

The intestinal absorption experiments were conducted by an *in-situ* closed loop method [46]. Male S.D. rats (250–300 g) were fasted overnight before experiment with free access to water and anesthetized with sodium pentobarbital (32 mg/kg body weight i.p.). The rats were randomly divided into three groups: jejunum group, ileum group and colon group. The intestine was exposed through the midline abdominal incision. After the bile duct was ligatured, a segment of jejunum, ileum or colon(as long as about 7 cm) was isolated and flushed with phosphate buffered saline (PBS, pH = 7.4) and tied off at both ends to form a closed loop. The residual buffer was expelled with air. The distal part of the loop was inserted with polyethylene tubing, and then closed by clipping with a forceps. Taxol^®^ or PTX-loaded GA micelle solution (2 mL) kept at 37 °C, was introduced into the loop and the tubing was closed with forceps. 0.2 mL of blood was withdrawn from the subclavian vein at each given time intervals, and then placed into heparinized tubes and separated immediately by centrifugation (4000 rpm for 10 min). The obtained plasma was stored at −20 °C for analysis. The plasma was analyzed by the LC-MS/MS method as shown in Section 3.3.10.

#### 3.3.12. Statistical Analysis

Data were expressed as the mean ± S.D. of at least three experiments. Pharmacokinetic parameters such as the area under the concentration–time curve (AUC), total body clearance (CL) and half-life (t_1/2_) were calculated using a non-compartmental model by the practical pharmacokinetic program-WinNonlin (version 1.5, Pharsight, Mountain View, CA, USA). Statistical significance was tested by two-tailed Student’s t-test and One-Way ANOVA test. The *p* value < 0.05 was considered as statistical significance. All calculations were performed using SPSS^®^ statistical software program (SPSS^®^13, SPSS Inc, Chicago, CA, USA).

## 4. Conclusions

In this study, we successfully designed a novel formulation of PTX with GA, achieving approximate 90% encapsulation efficiency. On account of the amphiphilic characteristics of GA, the combination could form micelles in aqueous medium to enhance the solubility of PTX. The drug loading rate could reach up to 7.90%. The PTX-loaded GA micelles had a small size (<250 nm) with narrow size distribution. *In vitro* release study showed that PTX-loaded GA micelles could be considered as a delayed drug release system. Compared with Taxol^®^, PTX-loaded GA micelles demonstrated a notable improvement of oral bioavailability *in vivo*, which could be largely due to the enhancement of the PTX absorption in jejunum and colon intestine. The increased absorption of PTX may be attributed to a comprehensive effect of GA on the following aspects: the enhancement of PTX solubility, the nanosize drug delivery system, the inhibition of P-gp efflux and the inhibition on CYP3A. Unlike many other new dosage forms of PTX, of which various materials are still a long way to go to verify their clinical use, GA could be a very promising carrier for the oral delivery system of PTX, since GA itself has been used in clinic for years.
